# Impact of Initial Cardiology Telemedicine Evaluation on Follow-Up Visits for Common Conditions: Quasi-Experimental Study

**DOI:** 10.2196/73509

**Published:** 2025-08-05

**Authors:** Neil M Kalwani, Harrison Koos, Emily Kohn, Vijaya Parameswaran, Anica Oesterle, Marina Adrianzen, Febri Kurniawan, Lubna Qureshi, Rajesh Dash, Paul Heidenreich, David Scheinker, Fatima Rodriguez

**Affiliations:** 1 Cardiology Section, Medical Service, VA Palo Alto Health Care System, 3801 Miranda Avenue, Cardiology (111C), Palo Alto, CA, 94304, United States, 1 6504935000; 2 Division of Cardiovascular Medicine and the Cardiovascular Institute, School of Medicine, Stanford University, Stanford, CA, United States; 3 Department of Health Policy, School of Medicine, Stanford University, Stanford, CA, United States; 4 Department of Management Science and Engineering, School of Engineering, Stanford University, Stanford, CA, United States; 5 Department of Medicine, School of Medicine and Public Health, University of Wisconsin, Madison, WI, United States; 6 Stanford Prevention Research Center, School of Medicine, Stanford University, Stanford, CA, United States; 7 Stanford Health Care, Palo Alto, CA, United States; 8 Division of Pediatric Endocrinology & Diabetes, School of Medicine, Stanford University, Stanford, CA, United States; 9 Clinical Excellence Research Center, School of Medicine, Stanford University, Stanford, CA, United States; 10 Division of Hospital Medicine, School of Medicine, Stanford University, Stanford, CA, United States

**Keywords:** telemedicine, cardiology, cardiovascular disease, ambulatory care, health care utilization, health care delivery, symptom assessment, chronic disease

## Abstract

**Background:**

Telemedicine use has increased significantly in cardiology clinics, but the impact of initial telemedicine evaluation on total visit usage is unknown.

**Objective:**

This study aimed to determine the effect of initial telemedicine evaluation on the number of follow-up visits within 6 months for common cardiovascular conditions at an academic health system.

**Methods:**

Electronic health records data were extracted for general cardiology visits. New patient visits (NPVs) were included occurring from June 1, 2020, to May 31, 2023, for 10 common cardiovascular conditions—atrial fibrillation or flutter, chest pain, coronary artery disease, dyslipidemia, dyspnea, heart failure, hypertension, palpitations, preoperative evaluation, and syncope or dizziness. The effect of initial telemedicine versus in-person evaluation on follow-up visits within 6 months was assessed using a 2-stage least squares instrumental variable model with the proportion of clinician telemedicine use as the instrument and adjustment for patient and visit characteristics.

**Results:**

There were 5528 NPVs conducted by 40 general cardiology clinicians during the study period. The average patient age was 56 (SD 17.5) years, 54.2% (2998/5528) were female, 43.2% (2389/5528) were non-Hispanic White, 24.7% (1368/5528) were Asian, 13.8% (761/5528) were Hispanic, 34.4% (1904/5528) were on Medicare, and 13.2% (729/5528) were on Medicaid. Of the NPVs, 53.5% (2959/5528) were conducted via telemedicine (2814/5528, 50.9% via video and 145/5528, 2.6% via phone). Telemedicine use for NPVs ranged from 0% to 100% (N=40) across individual clinicians. The average number of follow-up visits was 57 visits per 100 patients within 6 months across all diagnosis groups. Patients receiving telemedicine NPVs were more likely to have telemedicine follow-up visits than those receiving in-person NPVs (1354/1619, 83.6% vs 680/1533, 44.4%). In the instrumental variable analysis, the impact of initial telemedicine evaluation differed by presenting condition. There was an increase in follow-up visits for patients with syncope or dizziness (29.8 visits/100 patients, 95% CI 6.4-53.1), palpitations (34.9 visits/100 patients, 95% CI 18.6-51.1), chest pain (36.9 visits/100 patients, 95% CI 18.5-55.2), and dyspnea (37.0 visits/100 patients, 95% CI 11.8-62.0). There was a decrease in follow-up visits for patients with coronary artery disease (−29.5 visits/100 patients, 95% CI −50.3 to −8.6) and dyslipidemia (−24.5 visits/100 patients, 95% CI −40.2 to −8.8). There was no significant effect for patients presenting for atrial fibrillation or flutter, heart failure, hypertension, and preoperative evaluation.

**Conclusions:**

The effect of initial telemedicine evaluation on follow-up visits varied significantly by presenting condition in this cardiology practice. Telemedicine use resulted in increased follow-up visits for patients presenting with symptomatic complaints, while for those presenting with chronic conditions, there was no significant effect or a decrease in visits. Future studies should assess strategies to target initial care modalities to appropriate patients in cardiology clinics with early in-person evaluation for symptomatic patients.

## Introduction

Telemedicine use remains above pre–COVID-19 pandemic levels, including in cardiology clinics, and patient comfort with telemedicine has increased substantially [1-5]. There is limited evidence, however, on when telemedicine can effectively substitute for in-person cardiovascular care and how telemedicine use affects overall care usage. These questions are particularly relevant given ongoing uncertainty about the future of telemedicine reimbursement and the concern that telemedicine may result in increased health care spending [6,7].

Telemedicine makes accessing care more convenient for patients, removing barriers such as the need to take time away from work or travel long distances to the clinic site, which may lead to increased visit usage [6,8]. Telemedicine could also increase downstream care if it is inadequate or inferior as compared with in-office care; for example, if an in-person physical examination is required [8]. Concerns have been raised about the quality of cardiovascular care provided through telemedicine with previous evaluations demonstrating that heart failure patients receiving telemedicine visits are less likely to have diagnostic testing or be prescribed guideline-directed medical therapies [9-11].

Studies examining the effect of telemedicine on visit usage in primary care practices have shown mixed results [4,12-16]. Most of these studies have directly compared outcomes for patients receiving initial telemedicine versus in-person care. This approach is subject to confounding from unobserved differences between telemedicine users and nonusers [7]. A limited number of studies have used quasi-experimental methods to more rigorously assess the impact of telemedicine on visit usage. These studies suggest that telemedicine use does not increase outpatient visits for some conditions, such as urinary tract infections and sinusitis, but may result in slightly higher visit usage overall [16-18]. These evaluations were limited to primary care conditions and used data from before or early in the COVID-19 pandemic, so the findings may not be generalizable to current efforts to implement telemedicine in cardiology practices.

Additional studies are needed employing rigorous observational study designs to evaluate the effect of telemedicine on downstream visit usage for patients with cardiovascular conditions. This evidence will be critical to guide future use of telemedicine by health systems and aid in the design of nationwide reimbursement policies.

We undertook a quality improvement study to assess the impact of initial telemedicine evaluation on follow-up visit usage for patients presenting with 10 common cardiovascular conditions at an academic health system. We hypothesized that the evaluation of new patients through telemedicine would not increase the total number of visits used by patients.

## Methods

### Setting

This study was undertaken at Stanford Health Care (SHC), a large academic health system based in Northern California. To continue providing care to patients during the COVID-19 pandemic, SHC rapidly transitioned visits to telemedicine (video and phone visits). Patients were able to access video visits via smartphone, tablet, or computer through the SHC electronic health record (EHR) patient portal application (Epic Systems). Clinicians accessed video visits through the EHR using VidyoConnect (Enghouse Vidyo, Inc). Clinicians at SHC were required to maintain appointment slots for new patient visits (NPVs). In June 2020, SHC returned to scheduling routine in-person visits, and clinicians could determine the proportion of their visits that would be conducted via telemedicine. When a new patient referral was received by the clinic, a new patient coordinator assigned the patient to the clinician with the next available NPV opening (either telemedicine or in-person). The coordinator then offered this appointment to the patient with the option to take a later appointment via an alternative modality with the same clinician if preferred. Follow-up visits were scheduled as determined by the patient and clinician during or after the first visit.

### Ethical Considerations

This study was deemed to be nonhuman subjects research by the Stanford institutional review board (protocol 67506). It was sponsored by the SHC Digital Health Care Integration team with the primary goal to assess the impact of telemedicine on care delivery at SHC. There was no informed consent or compensation for participants as this was a retrospective quality improvement study.

### Data

We extracted data on outpatient cardiology visits from January 1, 2017, to November 30, 2023, from the EHR. Data included patient demographics and visit characteristics, such as treating clinician and primary diagnosis.

The primary outcome of interest was the total number of follow-up visits completed by patients in the 6 months following their NPV. We defined follow-up visits as any visit occurring with a general cardiology clinician after the NPV, including visits that were anticipated and unanticipated at the time of the NPV. As a sensitivity analysis, we also assessed whether at least 1 follow-up visit was completed within 6 months of the NPV.

The primary study period included NPVs occurring from June 1, 2020, to May 31, 2023. Separately, we also examined NPVs in the prepandemic period between January 1, 2017, and August 31, 2019. We limited NPVs to those with primary visit diagnoses in 1 of 10 common general cardiology diagnosis groups: atrial fibrillation or flutter, chest pain, coronary artery disease, dyslipidemia, dyspnea, heart failure, hypertension, palpitations, preoperative evaluation, and syncope or dizziness (Multimedia Appendix 1). For each clinician, we calculated the fraction of their NPVs across these 10 diagnosis groups that were conducted via telemedicine during the primary study period. This value was used as a proxy for the availability of telemedicine NPV slots in their preset schedule. We limited our analysis to clinicians with a minimum of 10 NPVs across all diagnosis groups during the study period. We also excluded 2 clinicians who primarily practiced in a telemedicine-only program.

Our data included the following patient characteristics: age (<45 y, 45‐64 y, and ≥65 y), sex, race or ethnicity, preferred language, whether an interpreter was needed, insurance status (Medicare, Medicaid, private, and other), the natural logarithm of the distance between the patient’s home ZIP code and the clinic ZIP code (logarithmic scale used to normalize the data), and whether a fellow assisted the attending physician during the visit.

### Statistical Analysis

We compared baseline characteristics between patients receiving telemedicine and in-person NPVs using *t* tests for age, chi-square tests for categorical variables, and the Wilcoxon rank-sum test for distance to clinic. We also calculated the average number of follow-up visits during the study period by NPV modality and follow-up visit modality for each diagnosis group.

**Figure 1.**
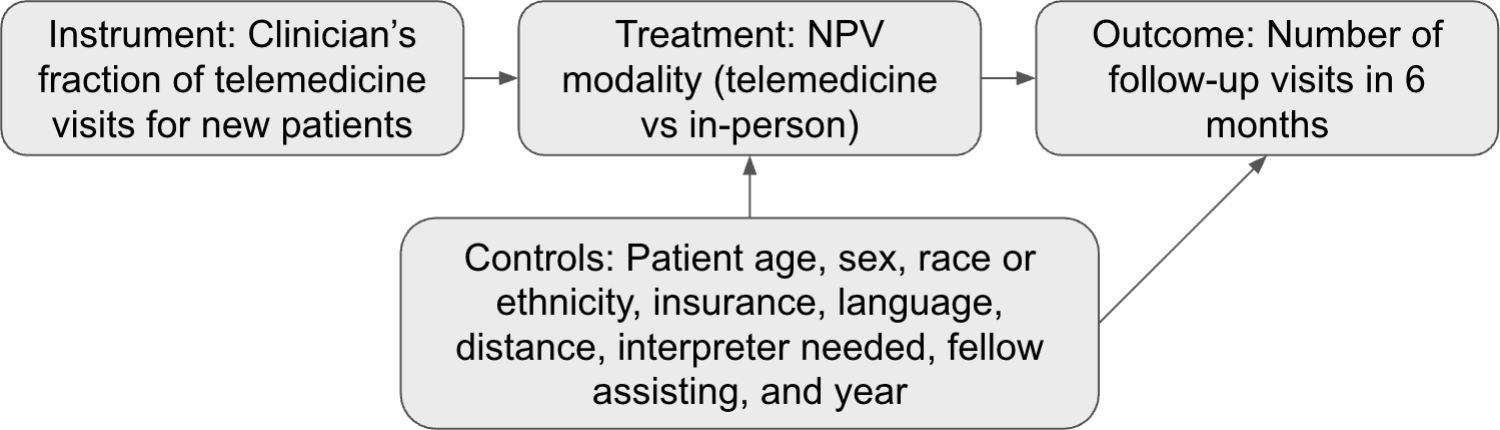
Instrumental variable model. NPV: new patient visit.

We used an instrumental variable model to estimate the causal effect of initial visit modality (telemedicine vs in-person) on follow-up visit usage within 6 months with the proportion of clinician telemedicine use for NPVs as the instrument (Figure 1). This approach requires that our instrument meets three conditions: (1) the instrument is associated with the treatment exposure (telemedicine NPV; relevance), (2) the instrument affects the outcome (follow-up visits) only through its effect on the treatment and not in any other direct way (exclusion restriction), and (3) the instrument was assigned to patients without regard to their potential outcomes (independence) [19].

For the first condition, we assumed that a patient’s NPV modality was influenced by the availability of appointment types for their assigned clinician and that these availabilities were captured by the clinician’s fraction of telemedicine NPVs during the primary study period. In other words, patients assigned to clinicians doing more telemedicine would be more likely to be offered a telemedicine visit. By construction, the instrument was correlated with treatment assignment.

We tested the second condition by regressing clinicians’ diagnosis-specific average rates of follow-up visits in the prepandemic period on their NPV telemedicine fraction during the primary study period, controlling for diagnosis group. If the second condition holds, there should be no significant association between telemedicine use in the primary study period and follow-up visit rates before the study period when telemedicine use was minimal. This analysis was limited to clinicians with at least 10 NPVs across all diagnosis groups in both the prepandemic and the primary study periods.

To test the third condition, we used prepandemic data to fit a linear regression model to predict the number of follow-up visits as a function of patient characteristics and diagnosis group. We used this model to predict the number of follow-up visits for each patient in the primary study period. We then assessed the association between the average predicted rate of follow-ups for each clinician’s patient panel and their NPV telemedicine fraction, controlling for diagnosis group. A lack of significant association would suggest that patients with higher predicted use of follow-up visits did not sort systematically to clinicians using more or less telemedicine.

For the primary analysis, we used 2-stage least-squares (2SLS) models to implement the instrumental variable analysis. We ran separate models for each of the 10 diagnosis groups including the covariates listed in Figure 1. We also ran 1 overall model that included data from all diagnosis groups with fixed effects for each group. The resulting estimates and 95% CIs with robust SEs were plotted along with the average number of follow-up visits for each diagnosis group. We reported the results scaled to 100 patients for ease of interpretation.

As a sensitivity analysis, we also ran the 2SLS model using the binary outcome of whether at least 1 follow-up visit took place within 6 months of the NPV (extensive margin) and reported the effect of telemedicine in terms of a linear probability model. Furthermore, we ran each model without covariates, except for the fixed effects for the diagnosis groups in the overall model. Finally, we ran a version of the model with the NPV modality interacted with time period (2022‐2023 vs 2020‐2021) to evaluate whether the effect of initial telemedicine evaluation changed significantly over time. *P* values <.05 were deemed to be statistically significant for all results.

## Results

There were 5528 general cardiology NPVs for the selected conditions between June 1, 2020, and May 31, 2023. Of these, 53.5% (2959/5528) were conducted via telemedicine (2814/5528, 50.9% via video, 145/5528, 2.6% via phone). These visits were delivered by 40 general cardiology clinicians with at least 10 NPVs across diagnosis groups. Visits for the included conditions represented 52.5% (5528/10,524) of all NPVs for these clinicians.

The average age of the patient cohort was 56 (SD 17.5) years with 54.2% (2998/5528) female, 43.2% (2389/5528) non-Hispanic White, 24.7% (1368/5528) Asian, and 13.8% (761/5528) Hispanic or Latino (Table 1). English was the preferred language for 86.3% (4769/5528) of patients, and 10.6% (588/5528) requested an interpreter to be used during their appointment. Private insurance was most common (2482/5528, 44.9%), followed by Medicare (1904/5528, 34.4%) and Medicaid (729/5528, 13.2%). The median distance between the patient and clinic ZIP code was 17.8 miles. Compared with patients who had an in-person NPV, those who received a telemedicine NPV were younger (mean age 53.57, SD 17.13 y vs 58.78, SD 17.60 y), more likely to be non-Hispanic White (1325/2959, 44.8% vs 1064/2569, 41.4%), more likely to have private insurance (1525/2959, 51.5% vs 957/2569, 37.3%), more likely to have English as their preferred language (2654/2959, 89.7% vs 2115/2569, 82.3%), and less likely to need an interpreter (220/2959, 7.4% vs 368/2569, 14.3%).

**Table 1.** Characteristics of patients receiving cardiology new patient visits, June 2020-May 2023.

Characteristic	New patient visits, n (%)			*P* value^a^
	Overall	Telemedicine	In-person	Telemedicine versus in-person
Overall	5528	2959 (53.5)	2569 (46.5)	—^b^
Age (y), mean (SD)	56.00 (17.50)	53.57 (17.13)	58.78 (17.60)	<.001
Sex				.63
Female	2998 (54.2)	1609 (54.4)	1389 (54.1)	
Male	2530 (45.8)	1350 (45.6)	1180 (45.9)
Race or ethnicity^c^				<.001
Asian	1368 (24.7)	700 (23.7)	668 (26)	
American Indian or Alaska Native	12 (0.2)	6 (0.2)	6 (0.2)
Black or African American	168 (3)	79 (2.7)	89 (3.5)
Hispanic or Latino	761 (13.8)	372 (12.6)	389 (15.1)
Native Hawaiian or Pacific Islander	55 (1)	32 (1.1)	23 (0.9)
White	2389 (43.2)	1325 (44.8)	1064 (41.4)
Unknown	775 (14)	445 (15)	330 (12.8)
Insurance				<.001
Private	2482 (44.9)	1525 (51.5)	957 (37.3)	
Medicare	1904 (34.4)	876 (29.6)	1028 (40)
Medicaid	729 (13.2)	333 (11.3)	396 (15.4)
Other	413 (7.5)	225 (7.6)	188 (7.3)
Preferred language				<.001
English	4769 (86.3)	2654 (89.7)	2115 (82.3)	
Spanish	214 (3.9)	75 (2.5)	139 (5.4)
Mandarin	137 (2.5)	50 (1.7)	87 (3.4)
Other	408 (7.4)	180 (6.1)	228 (8.9)
Interpreter needed				<.001
Yes	588 (10.6)	220 (7.4)	368 (14.3)	
No	4940 (89.4)	2739 (92.6)	2201 (85.7)
Distance to clinic (mi), median (IQR)	17.81 (9.19-31.00)	17.81 (9.19-33.29)	17.84 (9.19-29.51)	.11

a Categorical variables were compared with chi-square tests; age was compared with a *t* test; distance to clinic was compared with a Wilcoxon rank-sum test.

b Not applicable.

c All Hispanic or Latino patients were classified as “Hispanic or Latino.” Remaining patients are assumed to be non-Hispanic or Latino.

**Figure 2.**
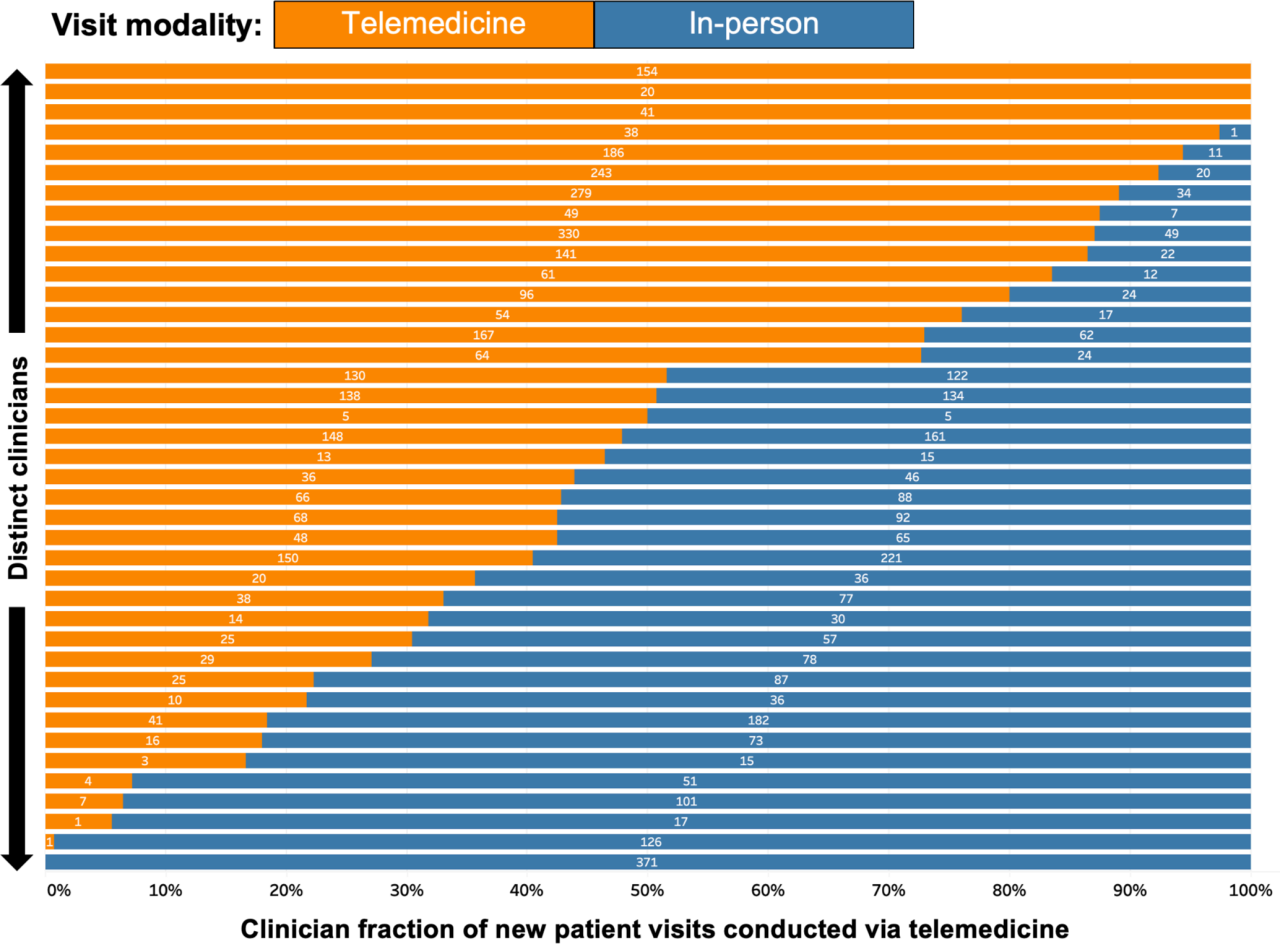
Variation in telemedicine use for new patient visits across cardiology clinicians. Numbers on the bars represent the count of visits of the given modality for the clinician in that row.

The preoperative evaluation diagnosis group had 21 distinct clinicians, while the remaining diagnosis groups all had 30-38 clinicians (Table 2). Among the diagnosis groups, preoperative evaluation had the lowest number of NPVs (n=106) and heart failure had the lowest telemedicine usage for NPVs (89/229, 38.9%), while dyslipidemia had the greatest number of NPVs (n=1187) and the highest telemedicine usage (884/1187, 74.5%). The overall level of telemedicine use for NPVs across all clinicians remained relatively consistent during the study period (Multimedia Appendix 2). Telemedicine use for individual clinicians varied widely from 0% to 100% (n=40; Figure 2).

**Figure 3.**
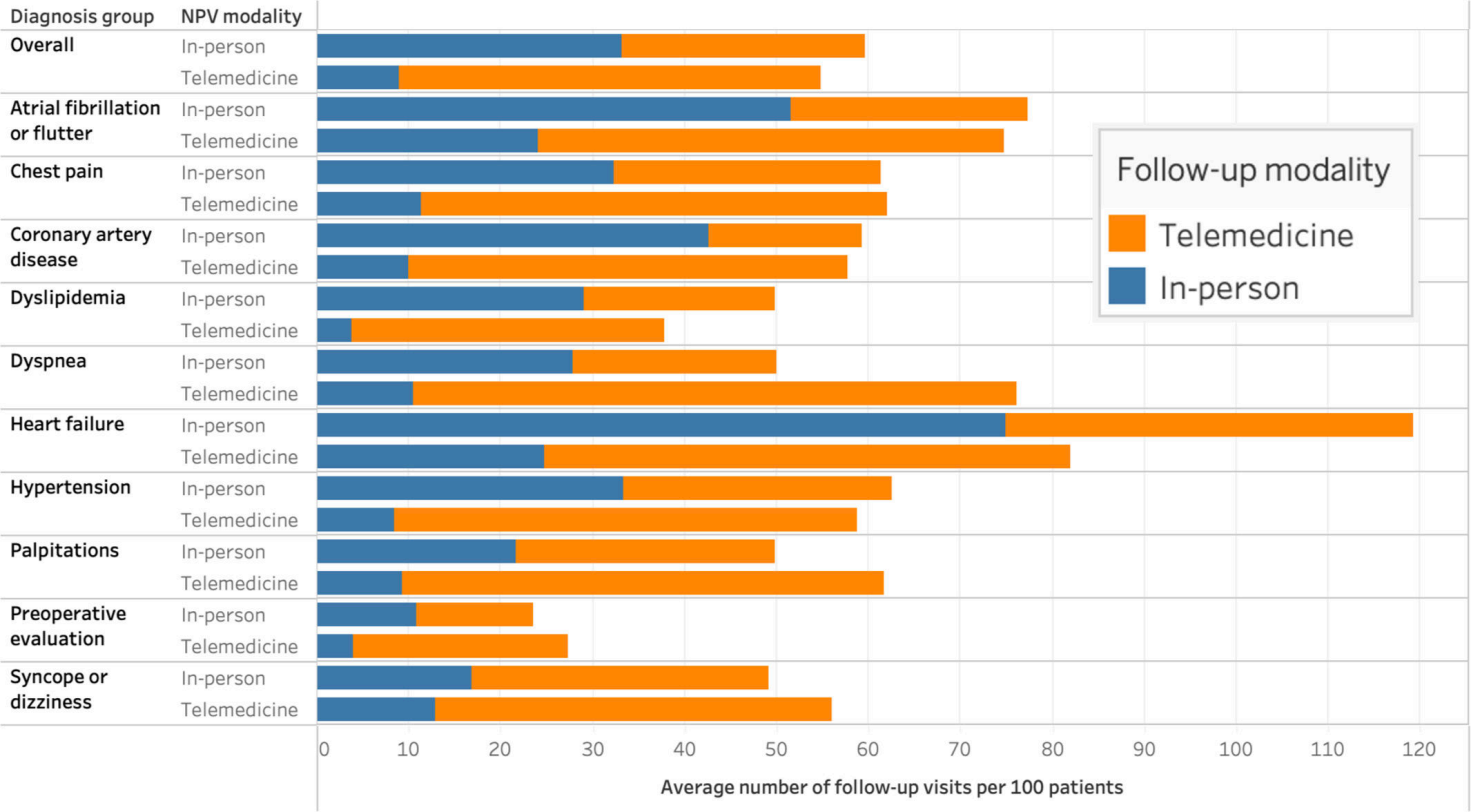
Average number of follow-up visits within 6 months by diagnosis group and new patient visit modality. NPV: new patient visit.

The average rate of follow-up visits within 6 months of an NPV was 57.0 visits/100 patients across all diagnoses, with diagnosis-specific rates ranging from 25.5 visits/100 patients for preoperative evaluation to 104.8 visits/100 patients for heart failure. Overall, patients receiving telemedicine NPVs had 83.6% (1354/1619) of their follow-up visits conducted via telemedicine, while those receiving in-person NPVs had 44.4% (680/1533) of their follow-up visits conducted via telemedicine. Within each diagnosis group, patients receiving telemedicine NPVs had more telemedicine follow-up visits and fewer in-person follow-up visits on average than patients receiving in-person NPVs (Figure 3). Patients received a majority of follow-up visits by the same modality as their NPV visit across all conditions, except for those receiving in-person NPVs for palpitations, preoperative evaluation, and syncope or dizziness, who received a majority of their follow-up visits by telemedicine.

**Table 2.** Characteristics of cardiology new patient visits across diagnosis groups, June 2020-May 2023.

Diagnosis Group	Total new patient visits, n	Telemedicine new patient visits, n (%)^a^	Clinicians, n
Overall	5528	2959 (53.5)	40
Atrial fibrillation or flutter	219	87 (39.7)	33
Chest pain	999	435 (43.5)	38
Coronary artery disease	618	322 (52.1)	37
Dyslipidemia	1187	884 (74.5)	37
Dyspnea	333	143 (42.9)	34
Heart failure	229	89 (38.9)	30
Hypertension	695	366 (52.7)	37
Palpitations	886	450 (50.8)	36
Preoperative evaluation	106	51 (48.1)	21
Syncope or dizziness	256	132 (51.6)	35

a Telemedicine % is calculated with respect to the overall number of new patient visits for the diagnosis group.

Clinicians’ rates of follow-up visits in the prepandemic period were not significantly associated with their overall fraction of NPVs delivered via telemedicine during the primary study period (*P*=.57; Multimedia Appendix 3). In addition, the average predicted follow-up rates for clinicians’ patient panels using prepandemic data were not significantly associated with their telemedicine use during the study period (*P*=.15; Multimedia Appendix 4).

**Figure 4.**
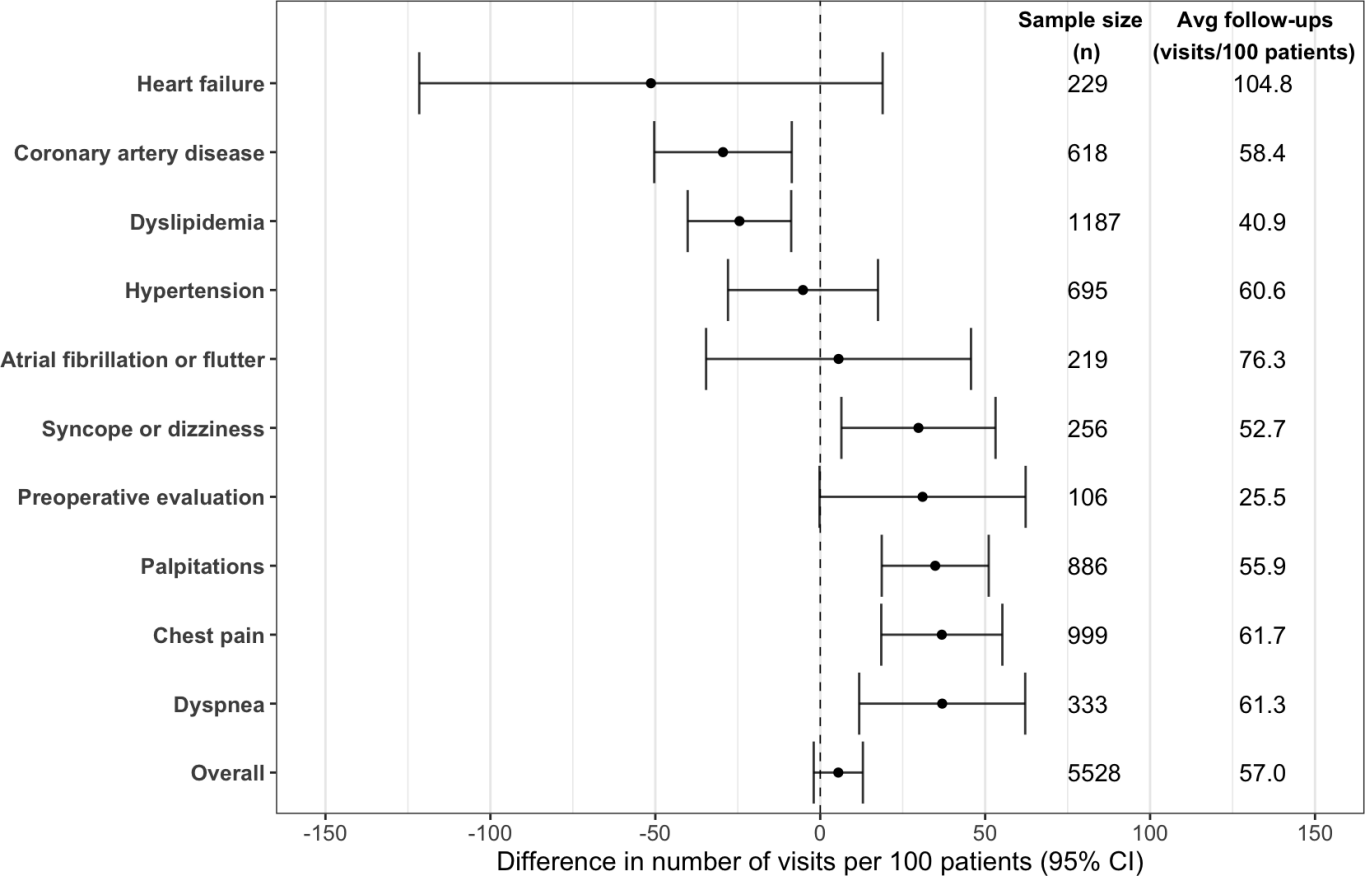
Effect of initial telemedicine versus in-person evaluation on follow-up visits within 6 months per 100 patients across diagnosis groups. The overall model includes data from each of the 10 diagnosis groups. 95% CIs are based on robust SEs. Avg: average.

Based on the 2SLS models, the impact of telemedicine use for NPVs on the number of follow-up visits within 6 months differed by diagnosis group (Figure 4). Telemedicine use for syncope or dizziness, palpitations, chest pain, and dyspnea resulted in significant increases in the number of follow-up visits with point estimates ranging from 29.8 visits/100 patients (95% CI 6.4-53.1) for syncope or dizziness to 37.0 visits/100 patients (95% CI 11.8-62.1) for dyspnea, while telemedicine use for coronary artery disease and dyslipidemia resulted in significant decreases in follow-up visits (−29.5 visits/100 patients, 95% CI −50.3 to −8.6 and −24.5 visits/100 patients, 95% CI −40.2 to −8.8, respectively; Multimedia Appendix 5). There was no significant effect of telemedicine use on follow-up visits for atrial fibrillation or flutter, heart failure, hypertension, and preoperative evaluation. The overall model including visits for all diagnosis groups showed a small increase in follow-up visits that was not statistically significant (5.5 visits/100 patients, 95% CI −2.0 to 12.9).

The 2SLS model estimating the effect of telemedicine evaluation on whether a follow-up visit occurred at all showed qualitatively similar results although the overall and preoperative evaluation groups showed statistically significant positive effects (MultimediaAppendices 6  7). When all control variables were dropped, the results were also qualitatively similar, except for the coronary artery disease group, for which the effect was marginally insignificant for both outcomes of interest (MultimediaAppendices 8  9). Finally, when an interaction term for NPV modality with time period (2022‐2023 vs 2020‐2021) was included, the effect of initial telemedicine evaluation on the number of follow-up visits was not significantly different between these periods for any of the diagnosis groups nor the overall model (Multimedia Appendix 10).

## Discussion

### Principal Findings

In this instrumental variable analysis, we found that telemedicine use for initial evaluation of cardiology patients had varying effects on the number of follow-up visits within 6 months across presenting conditions. For patients presenting for syncope or dizziness, palpitations, chest pain, and dyspnea, initial telemedicine evaluation increased the number of follow-up visits. For patients presenting for coronary artery disease and dyslipidemia, it decreased the number of follow-up visits. And for patients presenting for atrial fibrillation or flutter, heart failure, hypertension, and preoperative evaluation, there was no significant effect. To our knowledge, this is the first study using an instrumental variable approach to assess the impact of telemedicine on follow-up visit usage and the first examining this topic in a cardiology clinic setting.

As has been described previously, we found significant sociodemographic differences between patients receiving initial telemedicine versus in-person evaluation [20]. Patients receiving telemedicine NPVs were younger on average and were less likely to be Black or African American or Hispanic or Latino, to be covered by Medicaid, to have non–English-language preference, and to require an interpreter. These findings highlight persistent disparities in telemedicine use, which were evident even in this period including longer-term follow-up during the COVID-19 pandemic. Continued efforts are needed to broaden access to telemedicine across all patient groups.

The observed differences between patients receiving telemedicine and in-person care suggest a high likelihood of additional unobserved differences between these groups, for example, in illness severity, which limit attempts to compare outcomes between these groups directly. Studies using rigorous observational designs, such as the instrumental variable approach used in this analysis, are needed to address this selection bias. In this evaluation, we take advantage of pseudorandom care modality assignment due to an external factor, differences in clinician telemedicine adoption, to assess the impact of telemedicine on care patterns.

We found that initial telemedicine evaluation increased the number of follow-up visits for diagnosis groups representing symptomatic complaints (syncope or dizziness, palpitations, chest pain, and dyspnea), while there was no significant effect or decreased visit usage for diagnosis groups representing chronic conditions (coronary artery disease, dyslipidemia, atrial fibrillation or flutter, heart failure, and hypertension). Initial telemedicine evaluation may increase visit usage for patients with undifferentiated symptomatic complaints in several ways. First, it may be more difficult to evaluate these conditions remotely without a physical examination, necessitating additional in-person visits. For example, clinicians may need to assess for findings of heart failure on physical examination to complete a workup for dyspnea, or they may need to assess orthostatic vital signs to complete an evaluation for syncope or dizziness. In addition, patients with these conditions may require in-person diagnostic testing, such as an electrocardiogram or laboratory testing, and an in-person visit may have been scheduled to facilitate these tests. Alternatively, due to the limitations of remote evaluation, symptomatic patients may not have been adequately diagnosed or treated at the first visit. As a result, additional visits may have been required due to persistent patient symptoms, for example, if a new diagnosis of heart failure was missed without the additional data provided by physical examination. Finally, it is possible that these symptomatic patients chose to schedule additional visits due to the increased convenience afforded by telemedicine. In this case, these visits may represent high-value care if they allowed for improved disease management. Additional studies are needed examining clinical outcomes following telemedicine evaluation to determine the value of the care provided.

It is unclear how telemedicine evaluation may have decreased follow-up visit usage for coronary artery disease and dyslipidemia. Both conditions do not typically have physical exam findings requiring in-person assessment, and patients with these presenting complaints are often clinically stable, especially in the absence of symptoms. Management of these conditions centers around risk factor control through pharmacologic treatment and lifestyle change, and it is possible that both of these may be enhanced by telemedicine. For example, telemedicine may facilitate incorporation of caregivers into visits and allow for improved medication review when patients are in their home environments. In addition, patients with these conditions seen through telemedicine may have been more willing to delay subsequent routine visits because they assumed it would be easier to schedule unanticipated telemedicine follow-up visits if needed.

We found that patients receiving telemedicine NPVs received a greater number of telemedicine follow-up visits and fewer in-person follow-up visits on average as compared with patients receiving in-person NPVs. Put another way, the initial visit modality was “sticky” and persisted through follow-up care, likely because patients and clinicians had stable preferences on telemedicine use over time [21-23]. As a result, the exposure in this study included both the initial telemedicine visit modality and the higher probability that follow-up visits would be of the same modality due to the clinician’s preference for telemedicine. Since these exposures were linked, we were unable to determine if the effect of telemedicine was due to its use for the initial visit alone or also the increased likelihood that telemedicine would be used for follow-up care.

Although we found that the impact of telemedicine varied by presenting condition, when all conditions were included, telemedicine evaluation resulted in only a small nonsignificant increase in the total number of follow-up visits. This suggests that broad implementation of telemedicine for cardiology NPVs may not substantially increase total visit usage, although the effect in each clinic would likely depend on the mix of conditions seen.

The instrumental variable approach used in this study relied on key assumptions. We assumed that clinicians’ degree of telemedicine adoption was not associated with their baseline likelihood to schedule follow-up visits. In support of this assumption, we found that clinicians’ rates of follow-up visits in the prepandemic period before telemedicine adoption were not significantly associated with their telemedicine use during the study period. We also assumed that patients with a tendency to use more follow-up visits did not systematically sort to clinicians with higher or lower telemedicine use. In support of this assumption, we found that the predicted follow-up rates for clinicians’ patient panels were also not significantly associated with their telemedicine use.

Our results highlight the complexity of determining whether telemedicine visits represent high- or low-value care for patients undergoing initial evaluation. The answer likely depends on the specific clinical scenario in addition to other patient-level factors. Patients seen in cardiology clinic with symptomatic complaints may benefit from initial in-person evaluation to allow for physical examination and required same-day diagnostic testing. If an in-person visit cannot be scheduled in a timely fashion, however, telemedicine may still add value in spite of increasing total visits by allowing initial diagnostic testing and disease management to begin before in-person care can be arranged. For patients with chronic cardiovascular conditions, on the other hand, telemedicine may be superior to in-person care as it can facilitate greater access without the need to use valuable in-person clinic resources, and it may even reduce total visit usage for some conditions. Cardiology practices seeking to strategically incorporate telemedicine into routine care delivery should design new patient triage pathways that assign patients to initial visit modalities based on their presenting complaint, barriers to in-person care, and other clinical factors in addition to patient preference. Rigorous evaluation will be required to determine the impact of these pathways on care usage and clinical outcomes.

### Limitations

This project should be interpreted in the context of several limitations. First, this study was conducted using data from the outpatient cardiology clinics of a single academic health system, and the findings may not be generalizable to other settings and patient populations. Second, a significant portion of the data used in this study was from the COVID-19 pandemic when the relationship between telemedicine use and downstream care usage may not have been representative of long-term effects. Third, due to limitations of our dataset, we could not distinguish whether the effects of initial telemedicine evaluation were due to changes in the number of anticipated or unanticipated follow-up visits. Finally, we used an instrumental variable model to estimate the causal effect of telemedicine on follow-up visit usage, but we cannot be certain that the assumptions required for this approach were met. As noted above, we conducted analyses that supported these assumptions. We found that clinicians’ prepandemic rates of follow-up visits were not significantly associated with their telemedicine use during the study period, but it is possible that the pandemic affected practice patterns of clinicians who were high versus low telemedicine adopters differently, which could have biased our results. We also found that the predicted follow-up rates for clinicians’ patient panels based on patient characteristics were not significantly associated with clinician telemedicine use, but we were not able to include specific measures of illness severity or clinical complexity in this analysis to assess for patient sorting on the basis of these factors.

### Conclusions

In this study, we employed an instrumental variable approach to investigate the impact of initial telemedicine evaluation for common cardiovascular conditions on follow-up visit usage within 6 months. We found that the effect of telemedicine differed based on the presenting condition, with an increase in visits for patients presenting with symptomatic complaints and a decrease in visits or no effect for those presenting with chronic conditions. Future research should focus on evaluating strategies to target telemedicine to appropriate patients in cardiology clinics.

## Supplementary material

10.2196/73509 Multimedia Appendix 1 Diagnosis groups.

10.2196/73509 Multimedia Appendix 2 Fraction of new patient visits conducted via telemedicine, June 2020 to May 2023, based on our sample of 5528 new patient visits across 10 common outpatient cardiology diagnoses.

10.2196/73509 Multimedia Appendix 3 Association of clinicians’ prepandemic rate of follow-up visits with telemedicine use for new patient visits during the study period (exclusion restriction test). Sample size is 204 clinician-diagnosis group observations. The dependent variable is the average number of 6-month follow-up visits for patients seen by the clinician for the given diagnosis group between January 2017 and August 2019. The result of interest is the association between the clinicians’ fractions of new patient visits via telemedicine during COVID-19 and their pre–COVID-19 follow-up rates.

10.2196/73509 Multimedia Appendix 4 Association of the predicted rate of follow-up visits for clinicians’ patient panels with telemedicine use for new patient visits during the study period (independence test). Sample size is 338 clinician-diagnosis group observations. The dependent variable is the average predicted six-month follow-up visits for patients seen by the clinician for the given diagnosis group between June 2020 and May 2023. The prediction model is a linear regression based on patient characteristics and diagnosis, trained on data from January 2017 to August 2019. The result of interest is the association between the clinicians’ fractions of new patient visits via telemedicine during the COVID-19 pandemic and their patients’ predicted follow-up rates.

10.2196/73509 Multimedia Appendix 5 Regression table for the effect of initial telemedicine versus in-person evaluation on 6-month follow-up visits per 100 patients across diagnosis groups. Each estimate is based on a 2-stage least squares model fit on a different subset of data, split by diagnosis group. The overall model includes data from each of the 10 diagnosis groups. The estimated effect is the difference in follow-up visits for a patient receiving their new patient visit via telemedicine as opposed to in-person, scaled to 100 patients. All estimates were adjusted for age, race/ethnicity, preferred language, insurance, whether an interpreter was needed, the natural logarithm of the distance between the patient’s home ZIP code and the clinic ZIP code, whether a fellow assisted the attending physician during the visit, and year. The overall model included a control for diagnosis group. Robust standard errors are applied. Results correspond to Figure 4.

10.2196/73509 Multimedia Appendix 6 Effect of initial telemedicine versus in-person evaluation on the probability of having a follow-up visit within 6 months across diagnosis groups. Each estimate is based on a 2-stage least squares linear probability model fit on a different subset of data, split by diagnosis group. The overall model includes data from each of the 10 diagnosis groups. The estimated effect is the percentage point difference in likelihood of a patient receiving at least one follow-up visit within six months if their new patient visit is delivered via telemedicine as opposed to in-person. All estimates were adjusted for age, race/ethnicity, preferred language, insurance, whether an interpreter was needed, the natural logarithm of the distance between the patient’s home ZIP code and the clinic ZIP code, whether a fellow assisted the attending physician during the visit, and year. The overall model included a control for diagnosis group. 95% CIs are based on robust SEs.

10.2196/73509 Multimedia Appendix 7 Regression table for the effect of initial telemedicine versus in-person evaluation on the probability of having a follow-up visit within 6 months across diagnosis groups. Each estimate is based on a 2-stage least squares linear probability model fit on a different subset of data, split by diagnosis group. The overall model includes data from each of the 10 diagnosis groups. The estimated effect is the percentage point difference in likelihood of a patient receiving at least one follow-up visit within 6 months if their new patient visit is delivered via telemedicine as opposed to in-person. All estimates were adjusted for age, race/ethnicity, preferred language, insurance, whether an interpreter was needed, the natural logarithm of the distance between the patient’s home ZIP code and the clinic ZIP code, whether a fellow assisted the attending physician during the visit, and year. The overall model included a control for diagnosis group. Robust SEs are applied.

10.2196/73509 Multimedia Appendix 8 Regression table for the effect of initial telemedicine versus in-person evaluation on 6-month follow-up visits per 100 patients across diagnosis groups (sensitivity analysis with no controls). Each estimate is based on a 2-stage least squares model fit on a different subset of data, split by diagnosis group. The overall model includes data from each of the 10 diagnosis groups. The estimated effect is the difference in follow-up visits for a patient receiving their new patient visit via telemedicine as opposed to in-person, scaled to 100 patients. Estimates were not adjusted for any covariates, except for the overall model, which included a control for diagnosis group. Robust SEs are applied.

10.2196/73509 Multimedia Appendix 9 Regression Table for the Effect of Initial Telemedicine Versus In-Person Evaluation on the Probability of Having a Follow-Up Visit Within 6 Months Across Diagnosis Groups (Sensitivity Analysis with no Controls). Each estimate is based on a 2-stage least squares model fit on a different subset of data, split by diagnosis group. The overall model includes data from each of the 10 diagnosis groups. The estimated effect is the percentage point difference in likelihood of a patient receiving at least one follow-up visit within six months if their new patient visit is delivered via telemedicine as opposed to in-person. Estimates were not adjusted for any covariates, except for the overall model, which included a control for diagnosis group. Robust SEs are applied.

10.2196/73509 Multimedia Appendix 10 Regression table for the differential effect of initial telemedicine versus in-person evaluation on 6-month follow-up visits per 100 patients across diagnosis groups in 2022-2023 versus 2020-2021. Each estimate is based on a 2-stage least squares model fit on a different subset of data, split by diagnosis group. The overall model includes data from each of the 10 diagnosis groups. The estimated effect is the difference in follow-up visits for a patient receiving their new patient visit via telemedicine as opposed to in-person in 2022-2023 versus in 2020-2021 (ie, the interaction of new patient visit modality and period), scaled to 100 patients. All estimates were adjusted for age, race/ethnicity, preferred language, insurance, whether an interpreter was needed, the natural logarithm of the distance between the patient’s home ZIP code and the clinic ZIP code, whether a fellow assisted the attending physician during the visit, and year. The overall model included a control for diagnosis group. Robust SEs are applied.

## Abbreviations

2SLS: 2-stage least-squares
EHR: electronic health record
NPV: new patient visit
SHC: Stanford Health Care
