# Supplementary material for: Impact of Initial Cardiology Telemedicine Evaluation on Follow-Up Visits for Common Conditions: Quasi-Experimental Study
Source: J Med Internet Res. 2025 Aug 5;27:e73509. doi: 10.2196/73509 (PMC12330163; doi:10.2196/73509)
Supplement: Multimedia Appendix 1 [file jmir-v27-e73509-s001.docx]

**Multimedia Appendix 1.** Diagnosis Groups

| **Diagnosis Group** | **Specific Primary Visit Diagnoses** |
| --- | --- |
| Atrial Fibrillation / Flutter | AF (paroxysmal atrial fibrillation) (CMS-HCC); Atrial fibrillation and flutter (CMS-HCC); Atrial fibrillation with RVR (CMS-HCC); Atrial fibrillation, permanent (CMS-HCC); Atrial fibrillation, persistent (CMS-HCC); Atrial fibrillation, unspecified type (CMS-HCC); Atrial flutter, unspecified type (CMS-HCC); Atypical atrial flutter (CMS-HCC); Chronic atrial fibrillation (CMS-HCC); Longstanding persistent atrial fibrillation (CMS-HCC); New onset atrial fibrillation (CMS-HCC); Other persistent atrial fibrillation (CMS-HCC); PAF (paroxysmal atrial fibrillation) (CMS-HCC); Paroxysmal A-fib (CMS-HCC); Paroxysmal atrial fibrillation (CMS-HCC); Permanent atrial fibrillation (CMS-HCC); Persistent atrial fibrillation (CMS-HCC); Postoperative atrial fibrillation (CMS-HCC); S/P ablation of atrial fibrillation; Typical atrial flutter (CMS-HCC); Atrial fibrillation (CMS-HCC); Atrial fibrillation by electrocardiogram (CMS-HCC); Atrial fibrillation status post cardioversion (CMS-HCC); Atrial fibrillation with controlled ventricular response (CMS-HCC); Atrial fibrillation with rapid ventricular response (CMS-HCC); Atrial fibrillation with slow ventricular response (CMS-HCC); Atrial fibrillation, chronic (CMS-HCC); Atrial fibrillation, currently in sinus rhythm; Atrial fibrillation/flutter; Atrial flutter by electrocardiogram (CMS-HCC); Atrial flutter with rapid ventricular response (CMS-HCC); Atrial flutter, paroxysmal (CMS-HCC); H/O atrial flutter; History of atrial fibrillation; History of atrial flutter; Lone atrial fibrillation (CMS-HCC); Paroxysmal atrial fibrillation with RVR (CMS-HCC); Paroxysmal atrial flutter (CMS-HCC); S/P ablation of atrial flutter; Status post ablation of atrial fibrillation; Status post ablation of atrial flutter; Status post catheter ablation of atrial flutter |
| Chest Pain | Atypical chest pain; Chest discomfort; Chest pain, unspecified type; Chest pressure; Chest tightness; Chronic chest pain; Exertional chest pain; Other chest pain; Precordial pain; Chest pain on breathing; Chest pain on exertion; Chest pain, musculoskeletal; Nonspecific chest pain |
| Coronary Artery Disease | Atherosclerosis of native coronary artery of native heart without angina pectoris; CAD S/P percutaneous coronary angioplasty; Coronary artery disease involving coronary bypass graft of native heart without angina pectoris; Coronary artery disease involving native coronary artery of native heart with angina pectoris (CMS-HCC); Coronary artery disease involving native coronary artery of native heart with other form of angina pectoris (CMS-HCC); Coronary artery disease involving native coronary artery of native heart without angina pectoris; Coronary artery disease involving native coronary artery of native heart, angina presence unspecified; Coronary artery disease involving native heart without angina pectoris, unspecified vessel or lesion type; Coronary artery disease involving native heart, angina presence unspecified, unspecified vessel or lesion type; Coronary artery disease involving native heart, unspecified vessel or lesion type, unspecified whether angina present; Coronary artery disease of native artery of native heart with stable angina pectoris (CMS-HCC); Coronary artery disease with exertional angina (CMS-HCC); Coronary atherosclerosis due to calcified coronary lesion; Non-ST elevation myocardial infarction (NSTEMI) (CMS-HCC); NSTEMI (non-ST elevated myocardial infarction) (CMS-HCC); Old MI (myocardial infarction); S/P CABG (coronary artery bypass graft); Abnormal stress test; Atherosclerosis of native coronary artery of native heart with angina pectoris (CMS-HCC); Stable angina (CMS-HCC); Ischemia, myocardial, chronic; History of non-ST elevation myocardial infarction (NSTEMI); Hx of CABG; Atherosclerosis of coronary artery of native heart, angina presence unspecified, unspecified vessel or lesion type; Atherosclerosis of coronary artery of native heart, unspecified vessel or lesion type, unspecified whether angina present; Nonobstructive atherosclerosis of coronary artery; Atherosclerosis of native coronary artery of native heart, angina presence unspecified; Positive cardiac stress test; Atherosclerosis of coronary artery of native heart without angina pectoris, unspecified vessel or lesion type; Atherosclerosis of native coronary artery of native heart with stable angina pectoris (CMS-HCC); H/O heart artery stent; History of ST elevation myocardial infarction (STEMI); Hx of myocardial infarction; S/P CABG x 3; 3-vessel coronary artery disease; CAD in native artery; CAD of autologous artery bypass graft without angina; CAD, multiple vessel; Chronic coronary artery disease; Coronary artery disease due to calcified coronary lesion; Coronary artery disease due to lipid rich plaque; Coronary artery disease involving autologous artery coronary bypass graft without angina pectoris; Coronary artery disease involving autologous vein bypass graft, angina presence unspecified; Coronary artery disease involving autologous vein coronary bypass graft without angina pectoris; Coronary artery disease involving bypass graft of transplanted heart without angina pectoris; Coronary artery disease involving coronary bypass graft of native heart with angina pectoris (CMS-HCC); Coronary artery disease involving coronary bypass graft of native heart with other forms of angina pectoris (CMS-HCC); Coronary artery disease involving coronary bypass graft of native heart, angina presence unspecified; Coronary artery disease involving coronary bypass graft without angina pectoris, unspecified whether native or transplanted heart; Coronary artery disease involving native coronary artery of native heart with angina pectoris with documented spasm (CMS-HCC); Coronary artery disease involving native coronary artery of native heart with unstable angina pectoris (CMS-HCC); Coronary artery disease involving native coronary artery of native heart, unspecified whether angina present; Coronary artery disease involving native coronary artery without angina pectoris, unspecified whether native or transplanted heart; Coronary artery disease involving native coronary artery, angina presence unspecified, unspecified whether native or transplanted heart; Coronary artery disease involving native heart with angina pectoris and documented spasm, unspecified vessel or lesion type (CMS-HCC); Coronary artery disease involving native heart with angina pectoris, unspecified vessel or lesion type (CMS-HCC); Coronary artery disease involving native heart with other form of angina pectoris, unspecified vessel or lesion type (CMS-HCC); Coronary artery disease involving nonautologous biological coronary bypass graft with other forms of angina pectoris (CMS-HCC); Coronary artery disease involving other coronary artery bypass graft without angina pectoris; Coronary artery disease of autologous vein bypass graft with stable angina pectoris (CMS-HCC); Coronary artery disease of bypass graft of native heart with stable angina pectoris (CMS-HCC); Coronary artery disease of native heart with stable angina pectoris, unspecified vessel or lesion type (CMS-HCC); Coronary artery disease without angina pectoris, unspecified vessel or lesion type, unspecified whether native or transplanted heart; Coronary artery disease, angina presence unspecified, unspecified vessel or lesion type, unspecified whether native or transplanted heart; Coronary artery disease, non-occlusive; Hx of coronary artery disease; Mild coronary artery disease; Multiple vessel coronary artery disease; Non-occlusive coronary artery disease; Preclinical coronary artery disease; Triple vessel coronary artery disease |
| Dyslipidemia | Dyslipidemia; Elevated cholesterol; Familial hypercholesteremia; Familial hypercholesterolemia; Hypercholesteremia; Hypercholesterolemia; Hyperlipidemia, unspecified hyperlipidemia type; Mixed hyperlipidemia; Hypertriglyceridemia; High cholesterol; High triglycerides; Hypercholesterolemia with LDL greater than 190 mg/dL; Combined hyperlipidemia; Elevated LDL cholesterol level; Elevated triglycerides with high cholesterol; Elevated cholesterol with high triglycerides; Hyperlipidemia, mixed; Other hyperlipidemia; Pure hypercholesterolemia; Dyslipidemia (high LDL; low HDL); Dyslipidemia associated with type 2 diabetes mellitus (CMS-HCC); Dyslipidemia, goal LDL below 100; Dyslipidemia, goal LDL below 70; Mixed dyslipidemia |
| Dyspnea | Dyspnea, unspecified type; Dyspnea on exertion; Shortness of breath; DOE (dyspnea on exertion); Exertional shortness of breath; SOB (shortness of breath); Exertional dyspnea; SOB (shortness of breath) on exertion; Dyspnea and respiratory abnormality |
| Heart Failure | Acute on chronic combined systolic and diastolic heart failure (CMS-HCC); Acute on chronic diastolic heart failure (CMS-HCC); Acute on chronic systolic congestive heart failure (CMS-HCC); Acute on chronic systolic heart failure (CMS-HCC); Acute systolic heart failure (CMS-HCC); Cardiomyopathy due to chemotherapy (CMS-HCC); Cardiomyopathy, primary (CMS-HCC); Cardiomyopathy, unspecified type (CMS-HCC); Chemotherapy induced cardiomyopathy (CMS-HCC); Cardiomyopathy (CMS-HCC); Left ventricular systolic dysfunction; Chronic combined systolic (congestive) and diastolic (congestive) heart failure (CMS-HCC); Chronic combined systolic and diastolic congestive heart failure (CMS-HCC); Chronic combined systolic and diastolic heart failure (CMS-HCC); Chronic congestive heart failure, unspecified heart failure type (CMS-HCC); Chronic diastolic congestive heart failure (CMS-HCC); Chronic diastolic heart failure (CMS-HCC); Chronic heart failure with preserved ejection fraction (CMS-HCC); Chronic HFrEF (heart failure with reduced ejection fraction) (CMS-HCC); Chronic systolic (congestive) heart failure (CMS-HCC); Chronic systolic congestive heart failure (CMS-HCC); Chronic systolic heart failure (CMS-HCC); Chronic systolic HF (heart failure) (CMS-HCC); Congestive heart failure, unspecified HF chronicity, unspecified heart failure type (CMS-HCC); Diastolic congestive heart failure, unspecified HF chronicity (CMS-HCC); Dilated cardiomyopathy (CMS-HCC); Heart failure with preserved ejection fraction, unspecified HF chronicity (CMS-HCC); Heart failure with preserved left ventricular function (HFpEF) (CMS-HCC); Heart failure, systolic, chronic (CMS-HCC); Heart failure, unspecified HF chronicity, unspecified heart failure type (CMS-HCC); HFrEF (heart failure with reduced ejection fraction) (CMS-HCC); Idiopathic cardiomyopathy (CMS-HCC); Ischemic cardiomyopathy; Left heart failure (CMS-HCC); NICM (nonischemic cardiomyopathy) (CMS-HCC); Non-ischemic cardiomyopathy (CMS-HCC); Nonischemic cardiomyopathy (CMS-HCC); Other cardiomyopathy (CMS-HCC); Primary cardiomyopathy (CMS-HCC); Systolic heart failure, unspecified HF chronicity (CMS-HCC); (HFpEF) heart failure with preserved ejection fraction (CMS-HCC); ACC/AHA stage B heart failure with reduced ejection fraction (CMS-HCC); ACC/AHA stage B systolic heart failure due to ischemic cardiomyopathy (CMS-HCC); Acute combined systolic and diastolic congestive heart failure (CMS-HCC); Acute combined systolic and diastolic heart failure (CMS-HCC); Acute congestive heart failure, unspecified heart failure type (CMS-HCC); Acute diastolic CHF (congestive heart failure), NYHA class 3 (CMS-HCC); Acute diastolic heart failure (CMS-HCC); Acute on chronic clinical systolic heart failure (CMS-HCC); Acute on chronic combined systolic (congestive) and diastolic (congestive) heart failure (CMS-HCC); Acute on chronic combined systolic and diastolic CHF (congestive heart failure) (CMS-HCC); Acute on chronic combined systolic and diastolic congestive heart failure (CMS-HCC); Acute on chronic congestive heart failure, unspecified heart failure type (CMS-HCC); Acute on chronic diastolic CHF (congestive heart failure) (CMS-HCC); Acute on chronic diastolic congestive heart failure (CMS-HCC); Acute on chronic heart failure with preserved ejection fraction (CMS-HCC); Acute on chronic heart failure with reduced ejection fraction and diastolic dysfunction (CMS-HCC); Acute on chronic heart failure, unspecified heart failure type (CMS-HCC); Acute on chronic HFrEF (heart failure with reduced ejection fraction) (CMS-HCC); Acute on chronic right-sided heart failure (CMS-HCC); Acute on chronic systolic (congestive) heart failure (CMS-HCC); Acute systolic (congestive) heart failure (CMS-HCC); Acute systolic congestive heart failure (CMS-HCC); Biventricular heart failure (CMS-HCC); CHF (congestive heart failure), NYHA class I, chronic, diastolic (CMS-HCC); CHF (congestive heart failure), NYHA class I, chronic, systolic (CMS-HCC); CHF (congestive heart failure), NYHA class II, acute on chronic, combined (CMS-HCC); CHF (congestive heart failure), NYHA class II, chronic, systolic (CMS-HCC); CHF (congestive heart failure), NYHA class III, acute on chronic, systolic (CMS-HCC); CHF (congestive heart failure), NYHA class III, chronic, diastolic (CMS-HCC); CHF (congestive heart failure), NYHA class III, chronic, systolic (CMS-HCC); CHF due to valvular disease (CMS-HCC); Chronic clinical systolic heart failure (CMS-HCC); Chronic combined systolic and diastolic congestive heart failure, NYHA class 2 (CMS-HCC); Chronic diastolic (congestive) heart failure (CMS-HCC); Chronic diastolic CHF (congestive heart failure) (CMS-HCC); Chronic diastolic HF (heart failure) (CMS-HCC); Chronic heart failure with preserved ejection fraction (HFpEF) (CMS-HCC); Chronic heart failure with reduced ejection fraction and diastolic dysfunction (CMS-HCC); Chronic heart failure, unspecified heart failure type (CMS-HCC); Chronic left systolic heart failure (CMS-HCC); Chronic left ventricular systolic heart failure (CMS-HCC); Chronic left-sided CHF (congestive heart failure) (CMS-HCC); Chronic right-sided heart failure (CMS-HCC); Chronic systolic congestive heart failure due to valvular disease (CMS-HCC); Combined systolic and diastolic congestive heart failure, unspecified HF chronicity (CMS-HCC); Combined systolic and diastolic heart failure, unspecified HF chronicity (CMS-HCC); Congestive heart failure with LV diastolic dysfunction, NYHA class 1 (CMS-HCC); Diastolic CHF with preserved left ventricular function, NYHA class 2 (CMS-HCC); Diastolic dysfunction with chronic heart failure (CMS-HCC); Diastolic heart failure, unspecified HF chronicity (CMS-HCC); End stage heart failure (CMS-HCC); Heart failure and kidney disease due to high blood pressure (CMS-HCC); Heart failure of unknown type (CMS-HCC); Heart failure with normal ejection fraction (CMS-HCC); Heart failure with preserved ejection fraction (CMS-HCC); Heart failure with reduced ejection fraction (CMS-HCC); Heart failure, left systolic, chronic (CMS-HCC); Heart failure, left, with LVEF 31-40% (CMS-HCC); Hypertensive heart and kidney disease with acute on chronic diastolic congestive heart failure and stage 5 chronic kidney disease on chronic dialysis (CMS-HCC); Hypertensive heart and kidney disease with HF and with CKD stage III (CMS-HCC); Hypertensive heart disease with chronic combined systolic and diastolic congestive heart failure (CMS-HCC); Hypertensive heart disease with chronic systolic congestive heart failure (CMS-HCC); Left heart failure with left ejection fraction 30-40 percent (CMS-HCC); Left HF (heart failure) (CMS-HCC); Other heart failure (CMS-HCC); Right-sided heart failure, unspecified HF chronicity (CMS-HCC); Systolic congestive heart failure with reduced left ventricular function, NYHA class 2 (CMS-HCC); Systolic congestive heart failure, unspecified HF chronicity (CMS-HCC); Systolic dysfunction with acute on chronic heart failure (CMS-HCC); Systolic heart failure, chronic (CMS-HCC) |
| Hypertension | Benign essential hypertension; Essential hypertension; Secondary hypertension; Elevated blood pressure reading; Essential (primary) hypertension; Essential hypertension, benign; HTN (hypertension), benign; Hypertension, essential; Hypertension, unspecified type; Primary hypertension; Benign essential HTN; Benign hypertension; Essential hypertension with goal blood pressure less than 130/80; Essential hypertension with goal blood pressure less than 130/85; Essential hypertension with goal blood pressure less than 140/90; HTN (hypertension) with goal to be determined; HTN, goal below 140/90; Hypertension due to endocrine disorder; Hypertension secondary to other renal disorders; Hypertension with heart disease; Hypertension, benign; Hypertension, benign essential, goal below 140/90; Hypertension, essential, benign; Hypertension, malignant; Hypertensive cardiomyopathy, without heart failure (CMS-HCC); Hypertensive heart disease without congestive heart failure; Hypertensive heart disease, unspecified whether heart failure present; Labile essential hypertension; Labile hypertension; Malignant hypertension; Malignant hypertension with chronic kidney disease stage II; Renovascular hypertension; Resistant hypertension |
| Palpitations | Heart palpitations; Palpitations; History of palpitations; Intermittent palpitations; Palpitation |
| Preoperative Evaluation | Encounter for pre-operative cardiovascular clearance; Pre-op evaluation; Pre-op exam; Pre-op examination; Pre-operative cardiovascular examination; Pre-operative clearance; Pre-operative examination; Pre-transplant evaluation for liver transplant; Preop cardiovascular exam; Preop examination; Preoperative cardiovascular examination; Pre-op chest exam; Pre-op testing; Pre-operative exam; Pre-procedural examination; Pre-procedure lab exam; Preop testing; Preoperative clearance; Preoperative testing |
| Syncope / Dizziness | Syncope, unspecified syncope type; Syncope and collapse; Dizziness; Pre-syncope; Lightheadedness; Vasovagal syncope; Dizziness and giddiness; Postural dizziness with presyncope; Near syncope; Episodic lightheadedness; Lightheaded; History of syncope; Recurrent syncope |

NOTES: Patients were assigned to a given diagnosis group if the primary diagnosis for their new patient visit was listed in the table above
