# Supplementary figures and images for: Impact of Initial Cardiology Telemedicine Evaluation on Follow-Up Visits for Common Conditions: Quasi-Experimental Study
Source: J Med Internet Res. 2025 Aug 5;27:e73509. doi: 10.2196/73509 (PMC12330163; doi:10.2196/73509)

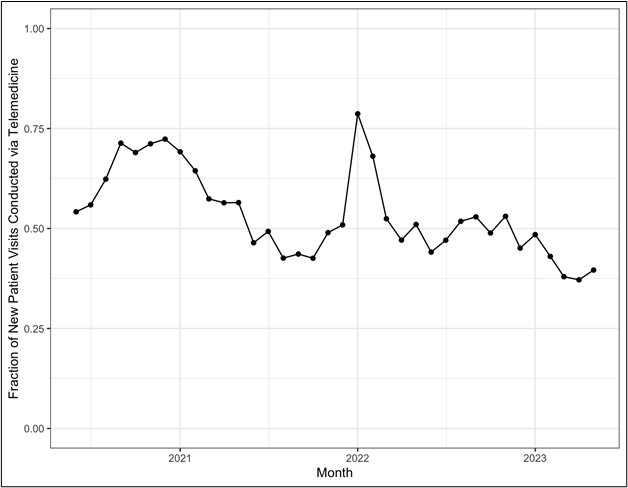

Supplement: Multimedia Appendix 2 [file jmir-v27-e73509-s002.png]

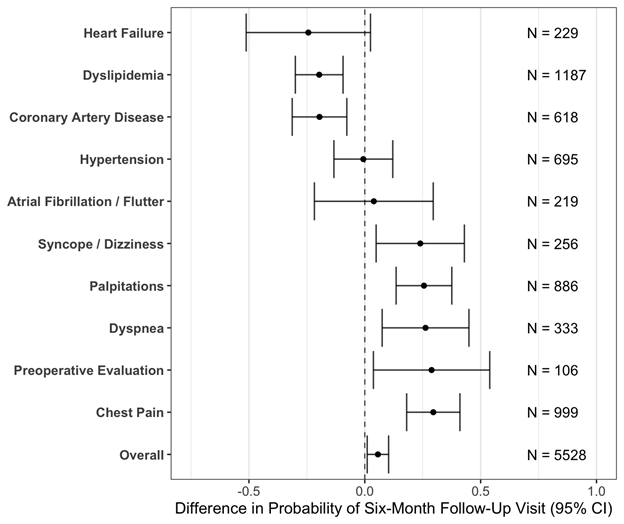

Supplement: Multimedia Appendix 6 [file jmir-v27-e73509-s006.png]
