# Supplementary material for: Impact of Initial Cardiology Telemedicine Evaluation on Follow-Up Visits for Common Conditions: Quasi-Experimental Study
Source: J Med Internet Res. 2025 Aug 5;27:e73509. doi: 10.2196/73509 (PMC12330163; doi:10.2196/73509)
Supplement: Multimedia Appendix 3 [file jmir-v27-e73509-s003.docx]

**Multimedia Appendix 3.** Association of Clinicians’ Pre-Pandemic Rate of Follow-Up Visits with Telemedicine Use for New Patient Visits During the Study Period (Exclusion Restriction Test)

| **Coefficient** | **Estimate** | **SE** | **95% CI** | **P Value** |
| --- | --- | --- | --- | --- |
| Intercept | 0.733 | 0.114 | (0.509, 0.956) | <.001 |
| Clinician Telemedicine Fraction | 0.051 | 0.089 | (-0.124, 0.227) | 0.569 |
| Chest Pain | -0.218 | 0.143 | (-0.498, 0.062) | 0.128 |
| Coronary Artery Disease | -0.092 | 0.144 | (-0.375, 0.191) | 0.525 |
| Dyslipidemia | -0.171 | 0.143 | (-0.451, 0.108) | 0.231 |
| Dyspnea | -0.079 | 0.144 | (-0.362, 0.203) | 0.583 |
| Heart Failure | 0.235 | 0.154 | (-0.067, 0.537) | 0.129 |
| Hypertension | -0.032 | 0.143 | (-0.312, 0.247) | 0.821 |
| Palpitations | -0.208 | 0.143 | (-0.488, 0.071) | 0.146 |
| Preoperative Evaluation | -0.486 | 0.150 | (-0.780, -0.193) | 0.001 |
| Syncope / Dizziness | -0.326 | 0.143 | (-0.606, -0.046) | 0.023 |

NOTES: Sample size is 204 clinician-diagnosis group observations. Dependent variable is the average number of six-month follow-up visits for patients seen by the clinician for the given diagnosis group between Jan 2017 - Aug 2019. The result of interest is the association between the clinicians’ fractions of new patient visits via telemedicine during COVID and their pre-COVID follow-up rates.
