# Supplementary material for: Impact of Initial Cardiology Telemedicine Evaluation on Follow-Up Visits for Common Conditions: Quasi-Experimental Study
Source: J Med Internet Res. 2025 Aug 5;27:e73509. doi: 10.2196/73509 (PMC12330163; doi:10.2196/73509)
Supplement: Multimedia Appendix 4 [file jmir-v27-e73509-s004.docx]

**Multimedia Appendix 4.** Association of the Predicted Rate of Follow-Up Visits for Clinicians’ Patient Panels with Telemedicine Use for New Patient Visits During the Study Period (Independence Test)

| **Coefficient** | **Estimate** | **SE** | **95% CI** | **P Value** |
| --- | --- | --- | --- | --- |
| Intercept | 0.912 | 0.010 | (0.894, 0.931) | <.001 |
| Clinician Telemedicine Fraction | -0.013 | 0.009 | (-0.030, 0.005) | 0.153 |
| Chest Pain | -0.274 | 0.012 | (-0.298, -0.251) | <.001 |
| Coronary Artery Disease | -0.139 | 0.012 | (-0.163, -0.116) | <.001 |
| Dyslipidemia | -0.406 | 0.012 | (-0.429, -0.382) | <.001 |
| Dyspnea | -0.188 | 0.012 | (-0.212, -0.164) | <.001 |
| Heart Failure | 0.136 | 0.013 | (0.111, 0.160) | <.001 |
| Hypertension | -0.140 | 0.012 | (-0.163, -0.116) | <.001 |
| Palpitations | -0.308 | 0.012 | (-0.332, -0.285) | <.001 |
| Preoperative Evaluation | -0.558 | 0.014 | (-0.585, -0.530) | <.001 |
| Syncope / Dizziness | -0.458 | 0.012 | (-0.482, -0.434) | <.001 |

NOTES: Sample size is 338 clinician-diagnosis group observations. Dependent variable is the average predicted six-month follow-up visits for patients seen by the clinician for the given diagnosis group between Jun 2020 - May 2023. The prediction model is a linear regression based on patient characteristics and diagnosis, trained on data from Jan 2017 - Aug 2019. The result of interest is the association between the clinicians’ fractions of new patient visits via telemedicine during COVID and their patients’ predicted follow-up rates.
