# Supplementary material for: Impact of Initial Cardiology Telemedicine Evaluation on Follow-Up Visits for Common Conditions: Quasi-Experimental Study
Source: J Med Internet Res. 2025 Aug 5;27:e73509. doi: 10.2196/73509 (PMC12330163; doi:10.2196/73509)
Supplement: Multimedia Appendix 5 [file jmir-v27-e73509-s005.docx]

**Multimedia Appendix 5.** Regression Table for the Effect of Initial Telemedicine Versus In-Person Evaluation on 6-Month Follow-Up Visits per 100 Patients Across Diagnosis Groups

| **Model** | **Estimate** | **SE** | **95% CI** | **P Value** | **Sample Size** |
| --- | --- | --- | --- | --- | --- |
| Overall | 5.47 | 3.80 | (-1.99, 12.92) | 0.151 | 5528 |
| Atrial Fibrillation / Flutter | 5.55 | 20.36 | (-34.61, 45.71) | 0.785 | 219 |
| Chest Pain | 36.86 | 9.35 | (18.50, 55.21) | <.001 | 999 |
| Coronary Artery Disease | -29.48 | 10.62 | (-50.33, -8.63) | 0.006 | 618 |
| Dyslipidemia | -24.52 | 8.00 | (-40.22, -8.83) | 0.002 | 1187 |
| Dyspnea | 36.97 | 12.79 | (11.81, 62.14) | 0.004 | 333 |
| Heart Failure | -51.33 | 35.63 | (-121.57, 18.92) | 0.151 | 229 |
| Hypertension | -5.23 | 11.58 | (-27.97, 17.51) | 0.652 | 695 |
| Palpitations | 34.86 | 8.26 | (18.64, 51.08) | <.001 | 886 |
| Preoperative Evaluation | 31.02 | 15.71 | (-0.22, 62.26) | 0.052 | 106 |
| Syncope / Dizziness | 29.78 | 11.86 | (6.41, 53.14) | 0.013 | 256 |

NOTES: Each estimate is based on a 2-stage least squares model fit on a different subset of data, split by diagnosis group. The overall model includes data from each of the 10 diagnosis groups. The estimated effect is the difference in follow-up visits for a patient receiving their new patient visit via telemedicine as opposed to in-person, scaled to 100 patients. All estimates were adjusted for age, race / ethnicity, preferred language, insurance, whether an interpreter was needed, the natural logarithm of the distance between the patient’s home ZIP Code and the clinic ZIP Code, whether a fellow assisted the attending physician during the visit, and year. The overall model included a control for diagnosis group. Robust standard errors are applied. Results correspond to Figure 4.
