# Supplementary material for: Impact of Initial Cardiology Telemedicine Evaluation on Follow-Up Visits for Common Conditions: Quasi-Experimental Study
Source: J Med Internet Res. 2025 Aug 5;27:e73509. doi: 10.2196/73509 (PMC12330163; doi:10.2196/73509)
Supplement: Multimedia Appendix 7 [file jmir-v27-e73509-s007.docx]

**Multimedia Appendix 7.** Regression Table for the Effect of Initial Telemedicine Versus In-Person Evaluation on the Probability of Having a Follow-Up Visit Within 6 Months Across Diagnosis Groups

| **Model** | **Estimate** | **SE** | **95% CI** | **P Value** | **Sample Size** |
| --- | --- | --- | --- | --- | --- |
| Overall | 0.057 | 0.024 | (0.010, 0.103) | 0.017 | 5528 |
| Atrial Fibrillation / Flutter | 0.039 | 0.130 | (-0.218, 0.295) | 0.765 | 219 |
| Chest Pain | 0.296 | 0.059 | (0.181, 0.411) | <.001 | 999 |
| Coronary Artery Disease | -0.195 | 0.060 | (-0.313, -0.078) | 0.001 | 618 |
| Dyslipidemia | 0.288 | 0.126 | (0.037, 0.539) | 0.025 | 1187 |
| Dyspnea | -0.197 | 0.052 | (-0.300, -0.094) | <.001 | 333 |
| Heart Failure | -0.244 | 0.136 | (-0.512, 0.025) | 0.075 | 229 |
| Hypertension | -0.006 | 0.065 | (-0.133, 0.121) | 0.922 | 695 |
| Palpitations | 0.255 | 0.061 | (0.135, 0.376) | <.001 | 886 |
| Preoperative Evaluation | 0.262 | 0.095 | (0.075, 0.450) | 0.006 | 106 |
| Syncope / Dizziness | 0.239 | 0.097 | (0.049, 0.430) | 0.014 | 256 |

NOTES: Each estimate is based on a 2-stage least squares linear probability model fit on a different subset of data, split by diagnosis group. The overall model includes data from each of the 10 diagnosis groups. The estimated effect is the percentage point difference in likelihood of a patient receiving at least one follow-up visit within six months if their new patient visit is delivered via telemedicine as opposed to in-person. All estimates were adjusted for age, race / ethnicity, preferred language, insurance, whether an interpreter was needed, the natural logarithm of the distance between the patient’s home ZIP Code and the clinic ZIP Code, whether a fellow assisted the attending physician during the visit, and year. The overall model included a control for diagnosis group. Robust standard errors are applied.
