# Supplementary material for: Impact of Initial Cardiology Telemedicine Evaluation on Follow-Up Visits for Common Conditions: Quasi-Experimental Study
Source: J Med Internet Res. 2025 Aug 5;27:e73509. doi: 10.2196/73509 (PMC12330163; doi:10.2196/73509)
Supplement: Multimedia Appendix 8 [file jmir-v27-e73509-s008.docx]

**Multimedia Appendix 8.** Regression Table for the Effect of Initial Telemedicine Versus In-Person Evaluation on 6-Month Follow-Up Visits per 100 Patients Across Diagnosis Groups (Sensitivity Analysis with no Controls)

| **Model** | **Estimate** | **SE** | **95% CI** | **P Value** | **Sample Size** |
| --- | --- | --- | --- | --- | --- |
| Overall | 5.62 | 3.70 | (6.29, 49.04) | 0.129 | 5528 |
| Atrial Fibrillation / Flutter | -0.02 | 17.89 | (-35.27, 35.23) | 0.999 | 219 |
| Chest Pain | 34.39 | 9.07 | (16.58, 52.20) | <.001 | 999 |
| Coronary Artery Disease | -18.25 | 10.60 | (-39.07, 2.58) | 0.086 | 618 |
| Dyslipidemia | -23.92 | 7.61 | (-1.20, 61.72) | 0.002 | 1187 |
| Dyspnea | 29.19 | 12.06 | (-38.84, -9.00) | 0.016 | 333 |
| Heart Failure | -43.63 | 35.02 | (-112.64, 25.38) | 0.214 | 229 |
| Hypertension | -1.85 | 11.96 | (-25.33, 21.62) | 0.877 | 695 |
| Palpitations | 35.93 | 7.69 | (20.83, 51.03) | <.001 | 886 |
| Preoperative Evaluation | 30.26 | 15.86 | (5.46, 52.92) | 0.059 | 106 |
| Syncope / Dizziness | 27.66 | 10.86 | (6.29, 49.04) | 0.011 | 256 |

NOTES: Each estimate is based on a 2-stage least squares model fit on a different subset of data, split by diagnosis group. The overall model includes data from each of the 10 diagnosis groups. The estimated effect is the difference in follow-up visits for a patient receiving their new patient visit via telemedicine as opposed to in-person, scaled to 100 patients. Estimates were not adjusted for any covariates, except for the overall model, which included a control for diagnosis group. Robust standard errors are applied.
