# Supplementary material for: Impact of Initial Cardiology Telemedicine Evaluation on Follow-Up Visits for Common Conditions: Quasi-Experimental Study
Source: J Med Internet Res. 2025 Aug 5;27:e73509. doi: 10.2196/73509 (PMC12330163; doi:10.2196/73509)
Supplement: Multimedia Appendix 9 [file jmir-v27-e73509-s009.docx]

**Multimedia Appendix 9.** Regression Table for the Effect of Initial Telemedicine Versus In-Person Evaluation on the Probability of Having a Follow-Up Visit Within 6 Months Across Diagnosis Groups (Sensitivity Analysis with no Controls)

| **Model** | **Estimate** | **SE** | **95% CI** | **P Value** | **Sample Size** |
| --- | --- | --- | --- | --- | --- |
| Overall | 0.060 | 0.023 | (0.015, 0.104) | 0.009 | 5528 |
| Atrial Fibrillation / Flutter | 0.029 | 0.118 | (-0.204, 0.262) | 0.807 | 219 |
| Chest Pain | 0.272 | 0.058 | (0.158, 0.386) | <.001 | 999 |
| Coronary Artery Disease | -0.102 | 0.059 | (-0.219, 0.015) | 0.086 | 618 |
| Dyslipidemia | -0.192 | 0.050 | (-0.289, -0.095) | <.001 | 1187 |
| Dyspnea | 0.198 | 0.093 | (0.015, 0.381) | 0.034 | 333 |
| Heart Failure | -0.227 | 0.133 | (-0.490, 0.035) | 0.089 | 229 |
| Hypertension | 0.033 | 0.063 | (-0.091, 0.157) | 0.598 | 695 |
| Palpitations | 0.273 | 0.057 | (0.161, 0.385) | <.001 | 886 |
| Preoperative Evaluation | 0.226 | 0.111 | (0.006, 0.446) | 0.044 | 106 |
| Syncope / Dizziness | 0.263 | 0.094 | (0.077, 0.448) | 0.006 | 256 |

NOTES: Each estimate is based on a 2-stage least squares model fit on a different subset of data, split by diagnosis group. The overall model includes data from each of the 10 diagnosis groups. The estimated effect is the percentage point difference in likelihood of a patient receiving at least one follow-up visit within six months if their new patient visit is delivered via telemedicine as opposed to in-person. Estimates were not adjusted for any covariates, except for the overall model, which included a control for diagnosis group. Robust standard errors are applied.
