# Supplementary material for: Impact of Initial Cardiology Telemedicine Evaluation on Follow-Up Visits for Common Conditions: Quasi-Experimental Study
Source: J Med Internet Res. 2025 Aug 5;27:e73509. doi: 10.2196/73509 (PMC12330163; doi:10.2196/73509)
Supplement: Multimedia Appendix 10 [file jmir-v27-e73509-s010.docx]

**Multimedia Appendix 10.** Regression Table for the Differential Effect of Initial Telemedicine Versus In-Person Evaluation on 6-Month Follow-Up Visits per 100 Patients Across Diagnosis Groups in 2022-2023 Versus 2020-2021

| **Model** | **Estimate** | **SE** | **95% CI** | **P Value** | **Sample Size** |
| --- | --- | --- | --- | --- | --- |
| Overall | -1.3 | 6.9 | (-14.7, 12.2) | 0.855 | 5528 |
| Atrial Fibrillation / Flutter | 11.5 | 35.3 | (-58.2, 81.2) | 0.745 | 219 |
| Chest Pain | -7.4 | 18.5 | (-43.7, 28.9) | 0.690 | 999 |
| Coronary Artery Disease | 34.6 | 22.3 | (-9.3, 78.5) | 0.122 | 618 |
| Dyslipidemia | 26.9 | 16.9 | (-6.3, 60.1) | 0.112 | 1187 |
| Dyspnea | -32.7 | 25.7 | (-83.4, 17.9) | 0.205 | 333 |
| Heart Failure | -63.2 | 62.1 | (-185.6, 59.2) | 0.310 | 229 |
| Hypertension | 14.5 | 22.7 | (-30.0, 59.0) | 0.521 | 695 |
| Palpitations | -21.9 | 16.4 | (-54.0, 10.2) | 0.181 | 886 |
| Preoperative Evaluation | -42.0 | 32.0 | (-105.7, 21.7) | 0.194 | 106 |
| Syncope / Dizziness | 1.0 | 24.9 | (-48.1, 50.2) | 0.967 | 256 |

NOTES: Each estimate is based on a 2-stage least squares model fit on a different subset of data, split by diagnosis group. The overall model includes data from each of the 10 diagnosis groups. The estimated effect is the difference in follow-up visits for a patient receiving their new patient visit via telemedicine as opposed to in-person in 2022-2023 versus in 2020-2021 (i.e. the interaction of NPV modality and period), scaled to 100 patients. All estimates were adjusted for age, race / ethnicity, preferred language, insurance, whether an interpreter was needed, the natural logarithm of the distance between the patient’s home ZIP Code and the clinic ZIP Code, whether a fellow assisted the attending physician during the visit, and year. The overall model included a control for diagnosis group. Robust standard errors are applied.
